# Deployable Laboratory Response to Influenza Pandemic; PCR Assay Field Trials and Comparison with Reference Methods

**DOI:** 10.1371/journal.pone.0025526

**Published:** 2011-10-12

**Authors:** Timothy J. J. Inglis, Adam J. Merritt, Avram Levy, Patricia Vietheer, Richard Bradbury, Adam Scholler, Glenys Chidlow, David W. Smith

**Affiliations:** 1 Division of Microbiology and Infectious Diseases, PathWest Laboratory Medicine WA, QEII Medical Centre, Nedlands, Western Australia, Australia; 2 3 Health Support Battalion, 17 Combat Support Services Brigade, Australian Army, Keswick, South Australia, Australia; 3 School of Pathology and Laboratory Medicine, Faculty of Medicine Dentistry and Health Science, University of Western Australia, Nedlands, Australia; 4 Viral Fusion Laboratory, Centre for Virology, Burnet Institute, Melbourne, Victoria, Australia; 5 School of Medicine, University of Tasmania, Hobart, Tasmania, Australia; 6 2 Health Support Battalion, 17 Combat Support Services Brigade, Australian Army, Enoggera, Queensland, Australia; 7 Pathology Laboratory, Rockhampton Base Hospital, Rockhampton, Queensland, Australia; University Hospital San Giovanni Battista di Torino, Italy

## Abstract

**Background:**

The influenza A/H1N1/09 pandemic spread quickly during the Southern Hemisphere winter in 2009 and reached epidemic proportions within weeks of the official WHO alert. Vulnerable population groups included indigenous Australians and remote northern population centres visited by international travellers. At the height of the Australian epidemic a large number of troops converged on a training area in northern Australia for an international exercise, raising concerns about their potential exposure to the emerging influenza threat before, during and immediately after their arrival in the area. Influenza A/H1N1/09 became the dominant seasonal variant and returned to Australia during the Southern winter the following year.

**Methods:**

A duplex nucleic acid amplification assay was developed within weeks of the first WHO influenza pandemic alert, demonstrated in northwestern Australia shortly afterwards and deployed as part of the pathology support for a field hospital during a military exercise during the initial epidemic surge in June 2009.

**Results:**

The nucleic acid amplification assay was twice as sensitive as a point of care influenza immunoassay, as specific but a little less sensitive than the reference laboratory nucleic acid amplification assay. Repetition of the field assay with blinded clinical samples obtained during the 2010 winter influenza season demonstrated a 91.7% congruence with the reference laboratory method.

**Conclusions:**

Rapid in-house development of a deployable epidemic influenza assay allowed a flexible laboratory response, effective targeting of limited disease control resources in an austere military environment, and provided the public health laboratory service with a set of verification tools for resource-limited settings. The assay method was suitable for rapid deployment in time for the 2010 Northern winter.

## Introduction

During the first few weeks of the 2009 influenza pandemic, infection spread quickly through New Zealand and Australia as winter was setting in. The World Health Organization influenza pandemic alert triggered the Australian Health Management Plan for Pandemic Influenza and set in motion a series of public health responses that included the World Health Organization Collaborating Centre for Influenza Reference and Research in Melbourne; National Influenza Centres in Melbourne, Sydney and Perth (PathWest Laboratory Medicine WA), and other reference laboratories in the Australian Public Health Laboratory Network. Influenza virus RNA extracts were obtained from the first cases confirmed in New Zealand and distributed to a group of regional reference laboratories, including our own, for in-house assay development. These assays were subsequently modified and validated on Australian clinical samples [1]. When the influenza pandemic arrived in Australia in May 2009, plans for a large multinational military exercise in northeastern Australia were at an advanced stage. We had previously used deployable PCR assays for emerging infectious disease response capacity-building in remote, resource limited settings overseas [2]. A deployable influenza A/H1N1/09 PCR assay was therefore added to the molecular diagnostic repertoire planned for the army exercise, and a preliminary proof-of-concept deployment to tropical Western Australia (WA) organized through the PathWest regional laboratory network [3]. Our concerns about spread of influenza among the military were based on the 1918-19 influenza pandemic which was thought to have had its origins in army training camps in the USA [4], when rapid spread of influenza was aided by large concentrations of service personnel in shared accommodation. Unlike in 1918, military health services now protect their personnel through influenza surveillance and vaccination programmes [5]. However, influenza A/H1N1/09 arrived in Australia several months before a vaccine was available. The arrival of an overseas contingent from an area already experiencing pandemic influenza to join the 2009 exercise placed an even greater emphasis on the need for clinical and public health laboratory support in the field. The only diagnostic method available in the field hospital was a point of care influenza A and B antigen detection ELISA (BD Directigen Flu A+B, Becton-Dickenson, VIC, Australia). This was backed up by referral of positive samples to the civilian health system for A/H1N1/09 PCR assay, a process that took 5–7 days to generate results due to the heavy workload at the regional hospital laboratory and its corresponding public health reference laboratory [6]. Early in the pandemic it was known that, compared to PCR, the point of care test (POCT) was insensitive for the detection of influenza, especially the pandemic strain [7]. We therefore set out to conduct an in-use evaluation of the field-deployable influenza PCR assay, then maintain its currency for deployment during future influenza epidemics.

## Materials and Methods

### Deployment logistics

Equipment items (thermocyclers, magnetic particle processor, microfuges, heating blocks, tube racks and pipettes) were shipped in air freight crates with secured moving parts, internal padding and dust exclusion measures. Consumables requiring cold chain during shipment were dispatched with dry ice or cold blocks as dictated by optimum transit temperature requirements. Nucleic acid amplification assays were dispatched as pre-dispensed mastermix, controls and ultra pure water in multiple small aliquots. Reagent stocks were calculated to allow 96 RNA purifications and nucleic acid reactions equivalent to 80 patient samples and appropriate controls.

### Clinical samples

These were collected within 48 hours of onset of illness from military personnel with clinically-suspected influenza. Plain cotton swabs were used to collect samples from both anterior nares and two swabs from the posterior oropharynx. Duplicate samples from all four locations were obtained for the POCT. A further series of 40 consecutive anonymous and previously analysed nasal swab samples was obtained through PathWest Laboratory Medicine WA during August 2010 in order to identify any changes in assay specificity. Clinical samples were collected and processed in accordance with standard Australian hospital laboratory practice as a field-deployed extension of the Public Health Laboratory Network influenza pandemic response. Standard pathology sample collection consent procedures were observed at all times and the process periodically reviewed by the field hospital's Senior Medical Officer. Collated results, analysis and this manuscript were reviewed by Defence Health commanders. Clearance for peer-review publication of the anonymised data was obtained through the office of the Colonel of Health, Forces Command, Australian Defence Force and from her subordinate commanders. Respiratory samples were obtained from both anterior nares of military personnel with suspected influenza, and twice from the posterior oropharynx using a pair of dedicated PCR swabs per patient. Duplicate samples from all four locations were obtained for influenza immunoassay.

### PCR assays

**Figure 1:**
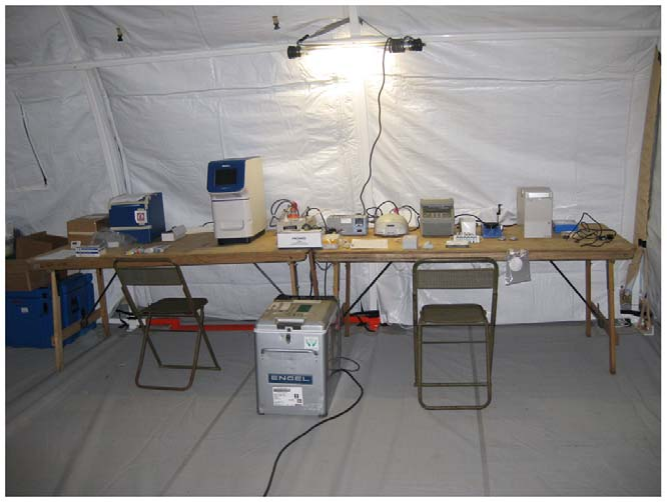
Deployable molecular microbiology laboratory. Deployable molecular biology equipment used during the exercise. The layout corresponds to the two modules; left table – static field hospital used for real time PCR assays, right table – field portable. Only the hand-held magnetic bead extraction device from the field portable module was used in an attempt to detect influenza A by PCR assay during the military exercise.

The reverse transcription PCR (RT-PCR) assays were performed on one of the deployable molecular laboratory main modules assembled to operate in a static field hospital setting (Figure 1). This comprised an automated magnetic particle processor for nucleic acid purification (MagMax-24, Applied Biosystems), a real time thermal cycler (StepOne, Applied Biosystems) and a laptop computer running under Windows XP. The influenza assay incorporated primers and probes directed at targets in the matrix gene of influenza A and in the HA gene of A/H1 2009 (table 1) that had been validated in an in-house duplex real time RT-PCR assay [1]. A comparison of viral RNA extraction methods was performed using the hand-held magnetic bead extraction device (6 tube magnetic rack, Applied Biosystems) from a second field laboratory module comprising more easily transportable equipment. The other components of that module (bench top microfuge, a conventional thermal cycler (2720, Applied Biosystems) and a labchip bioanalyser (Expert 2100, Agilent) were not used for the RT-PCR assays.

**Table 1:** Nucleic acid amplification assay primers & probes.

Target	Oligo name	Sequence (5′ to 3′)
Influenza A M gene†	INFLUA-MATF	CTTCTAACCGAGGTCGAAACGTA
	INFLUA-MATR	GGTGACAGGATTGGTCTTGTCTTTA
	FA-MAT-PR-FAM	FAM-TCAGGCCCCCTCAAAGCCGAG-BHQ1
H1N1 2009 HA gene	SWHA-F1.2	AAGGTGTAACGGCAGCATGTC
	SWHA-R1.2	TAGGATTTGCTGAGCTTTGGGTAT
	SWHA-probe 1.2	X-TGCTGGAGCAAAAAGCTTCT-MGBNFQ

† - Oligo sequences are as previously reported 2010 (1).

X- FAM was used for the monoplex H1 2009 assay, VIC was used for the duplex assay.

### Point of care tests (POCT)

Use of the Directigen Flu A+B immunoassay continued after the deployable nucleic acid amplification assays had been set up in the field and was run in parallel with PCR assays, the duplicate nasal and throat swabs being collected for this purpose. The tests were conducted in accordance with the manufacturer's instructions and were performed concurrently by different operators than those carrying out the PCR assays. Due to the short turnaround time of the POCT these results were available earlier than the PCR assay results, but results for the different tests were collected and interpreted separately.

### PCR assay sensitivity and specificity

This was initially determined for the StepOne thermocycler using serial dilutions of RNA extracts from samples known to be positive for the pre-pandemic seasonal influenza A/H1N1, influenza A/H1N1 2009, influenza A/H3N2 and influenza B. This preceded the field trial in 2009. The sensitivity and specificity of the influenza A RT-PCR assays, including the extraction and detection components, were reassessed in August 2010 using 40 consecutive influenza positive clinical samples to PathWest Laboratory Medicine WA and 8 negative controls (molecular grade ultrapure water, Fisher Biotech, WA), blind to parallel tests conducted with the reference laboratory high throughput equipment [1].

Virus isolation was not practicable under field conditions, and could not be performed at the reference laboratory due to workload constraints during the pandemic, and because prolonged storage and transit times meant that the viability of the virus could not be ensured.

## Results

Initial evaluations of the StepOne on serial dilutions of RNA extracts from known positive samples showed the matrix assay to be as sensitive as the reference assay for the detection of influenza A matrix gene of pre-pandemic seasonal influenza A/H1N1, influenza A/H1N1 2009 and influenza A/H3N2; and for the HA gene of influenza A/H1N1 2009 (Table 2). As expected it did not detect the matrix gene of influenza B. The A/H1N1 HA gene assay was also equivalent to the reference method for detection of that gene, but as expected did not detect the HA gene of any of the other influenza viruses. The reference method had a known limit of detection of 223–297 copies/ml for the assays used [1].

**Table 2:** Performance of deployable assay for detection of influenza A/H1N1 2009 according to specimen type and viral RNA extraction method.

	PoCT	MagMAX-24 extraction StepOne thermal cycler Labeled probe		6-tube hand-held extraction StepOne thermal cycler Labeled probe	
	Nose	Nose	Throat	Nose	Throat
Positive	7	12	11	4	3
Negative	6	0	0	4	2
Equivocal	0	0	1	0	0
Inhibitory	0	0	0	3	5
Not Done	0	0	0	2	3

POCT  =  point of care test.

A total of 12 patients were sampled in the field hospital during the 2009 exercise, resulting in 13 sets of swabs (one patient was sampled twice due to progression of symptoms following an initial negative result). The field laboratory influenza A RT-PCR assay with the combination of automated RNA extraction and real time PCR produced the highest diagnostic yield, with at least one A/H1N1/09 positive result from every referred patient. This was achieved in around three hours from swab collection. The assay using extracts from the handheld magnetic bead extraction device was less sensitive (nasal swab extract, 50%; throat swab extract, 60%), and some of the false negative results coincided with demonstrable PCR inhibition (3 nose, 5 throat swabs, respectively). The sensitivity of the hand held device extraction method was similar to the POCT which only detected around half the A/H1N1/09 results from nose swab extracts (54%), though the POCT had the additional disadvantage of not being able to determine the influenza virus subtype. The 50 µL sample volume required by the smaller portable magnetic bead extraction device (MagMax-24) goes some way to explain a lower sensitivity than we obtained with the high throughput magnetic bead extraction device (MagMax-96) which operates with a 200 µL sample input volume.

The deployable influenza nucleic acid amplification assay was again assessed in 2010 order to measure assay sensitivity against the influenza A viruses circulating in that year (Table 3). It was carried out blind to the positive clinical samples and water controls supplied by the reference laboratory The PCR assay achieved a sensitivity of 89.2% for detection of influenza A matrix gene and 93.5% for the A/H1N1 2009 HA gene in H1N1 clinical samples, while the specificity for both assays was 100%. Two of the false negative results (one A/H1N1/09 and influenza A matrix gene) were borderline results that were consistent with low viral titre in the clinical sample and favoured the higher volume reference laboratory assay. One sample was positive in the A/H1N1/09 assay but negative in the influenza A matrix assay which may be due to a slightly higher sensitivity of the A/H1N1/09 assay compared to the A/matrix assay. The other discrepant result was a detection failure on both the matrix and A/H1N1/09 field assays when the corresponding reference assay produced a low CT (high titre) result, possibly indicating a pipetting error.

**Table 3:** Deployable assay performance with 2010 clinical samples.

	Assay results	
Assay target	Influenza A matrix gene	Influenza A/H1N1 HA gene
Influenza A/H1N1 2009	28/31	29/31
Influenza A/H3	5/6	0/6
Influenza B	0/3	0/3
Negative controls	0/8	0/8
Sensitivity	89.2%	93.5%
Specificity	100%	100%
Positive predictive value	1.00	1.00
Negative predictive value	0.73	0.895
Total true positives	37	31
Total true negatives	11	17

Samples from 40 consecutive influenza-positive patients (31 influenza A/H1N1/09, 6 influenza A/H3 and 3 influenza B) plus 8 negative controls (ultrapure water).

## Discussion

We have shown that by adapting assays developed in a reference laboratory, it is possible to deliver a PCR-based assay for detection of a newly emerging infectious disease threat in field conditions, using test platforms currently in use.

The process of assay development described here was driven by sudden changes in the regional epidemiology of influenza [6], [8]. It relied on close collaboration with a network of public health laboratories, coupled with prior experience of field deployable molecular assays [1]–[3]. The approach we took to diagnostic and public health support aimed to provide specific results in a short enough time frame to influence decisions on treatment, infection control and health risk assessment. The ability to inform health decision-makers with specific laboratory results allowed more targeted use of oseltamivir and more effective use of a tented isolation ward [9]. Influenza spread among deployed military units can be rapid, as occurred in the spring of 1918 [4]. Several other instances of rapid spread of influenza in a military setting were reported following the 2009 pandemic, emphasizing the vulnerability of large formations of unvaccinated service personnel [10], [11].

The very high positive rate among the soldiers who were tested suggests that there was a larger number of other personnel with influenza who did not present to their regimental medical post, possibly due to the mild nature of the infection in this physically fit, young adult population. That would be consistent with the large numbers of civilian cases of pandemic influenza detected at that time by hospital and public health laboratories in Queensland and other parts of Australia due to the spread of infection beyond its initial Australian epicentre in Victoria [8]. The low sensitivity of POCT diagnostic aids for influenza has been reported previously both before and during the current pandemic, emphasising the importance of PCR assays for reliable diagnosis [7], [12], [13]. It is possible that the low sensitivity of the POCT used prior to deployment of the molecular laboratory led to low expectations of the field hospital's diagnostic service and therefore reluctance to submit further diagnostic samples from referring units. The modular laboratory approach allowed a degree of adaptation to these changing priorities, and was limited only by the scope and range of the reagent stock. The static field hospital module was able to meet variable levels of demand (from 1 to 24 samples per batch) for nine days without resupply. It was able to cope with basic quality control and troubleshooting during the initial insertion and operational phases including determination of extraction efficiency. The equipment was surprisingly robust and tolerated extreme conditions of varying temperature, vibration and dust.

The RT-PCR field assay using automated viral RNA extraction appeared to be slightly less sensitive than the tests performed by the reference method in the 2010 evaluation, possibly due to the lower specimen volume used (50 µL compared with 200 µL) and ideally this needs be addressed. However, it performed well in our small number of patients and was clearly superior to the POCT in the 2009 field trial, though larger sample numbers are required to properly assess the diagnostic sensitivity and specificity. Also, it would be preferable to include specific assays for influenza A/H3 and for influenza B to improve the diagnostic yield. It was also apparent that there was a trade-off between sensitivity and portability, since the highly mobile laboratory module (hand held magnetic bead extraction device) did not perform with a higher sensitivity than the POCT. Further work is needed to develop an acceptable near-patient test.

There are an increasing number of commercially available systems for simplified PCR testing for influenza viruses [14] at various stages of development and validation that may be able to fulfill similar roles. Some are designed for possible use outside large laboratories [15] but have not been tested in field laboratories. Others have the potential to deliver results closer to the patient [14], [16] but have yet to be evaluated in that setting for humans, though there is some early data from animal studies [17] . The weaknesses of all these devices is that they are not easily or quickly adaptable to new or changing pathogens, they may not offer the range of tests required in different geographic or climatic situations, and they may be expensive to perform.

A deployable influenza assay capability lends itself to temporary operation in small hospitals where the faster turn around times of close to point of care tests can be exploited to maximum effect. We have shown that a deployable laboratory response can be mounted rapidly and operated in austere, resource limited settings to meet an anticipated surge of epidemic influenza cases shortly after the onset of a pandemic. Preparation of a deployable laboratory capability based on influenza virus strains circulating during the southern hemisphere winter may enhance the public health laboratory response to influenza. Furthermore we believe this approach has applications for the detection of a wide range of infectious agents in similar settings, and can be tailored to meet local requirements.
